# Food insecurity and linear growth of adolescents in Jimma Zone, Southwest Ethiopia

**DOI:** 10.1186/1475-2891-12-55

**Published:** 2013-05-02

**Authors:** Tefera Belachew, David Lindstrom, Craig Hadley, Abebe Gebremariam, Wondwosen Kasahun, Patrick Kolsteren

**Affiliations:** 1 Department of Population and Family Health, Nutrition Unit, College of Public Health and Medical Sciences, Jimma University, PO.Box:1104, Jimma, Ethiopia; 2 Department of Food Safety and Food Quality, Faculty of Bioscience Engineering, Ghent University, Coupure Links 653, B- 9000 Ghent, Belgium; 3 Department of Sociology, Brown University, Box 1916, Providence, RI 02912 USA; 4 Department of Anthropology, Emory University, 207 Anthropology Building 1557 Dickey Drive, Atlanta, USA; 5 Department of Epidemiology and Biostatistics, College of Public Health and Medical Sciences, Jimma University, PO.Box:1104, Jimma, Ethiopia; 6 Nutrition and Child Health Unit, Department of Public Health, Institute of Tropical Medicine, Nationalestraat 155, 2000 Antwerpen, Belgium

## Abstract

**Background:**

Although many studies showed that adolescent food insecurity is a pervasive phenomenon in Southwest Ethiopia, its effect on the linear growth of adolescents has not been documented so far. This study therefore aimed to longitudinally examine the association between food insecurity and linear growth among adolescents.

**Methods:**

Data for this study were obtained from a longitudinal survey of adolescents conducted in Jimma Zone, which followed an initial sample of 2084 randomly selected adolescents aged 13–17 years. We used linear mixed effects model for 1431 adolescents who were interviewed in three survey rounds one year apart to compare the effect of food insecurity on linear growth of adolescents.

**Results:**

Overall, 15.9% of the girls and 12.2% of the boys (P=0.018) were food insecure both at baseline and on the year 1 survey, while 5.5% of the girls and 4.4% of the boys (P=0.331) were food insecure in all the three rounds of the survey. In general, a significantly higher proportion of girls (40%) experienced food insecurity at least in one of the survey rounds compared with boys (36.6%) (P=0.045).

The trend of food insecurity showed a very sharp increase over the follow period from the baseline 20.5% to 48.4% on the year 1 survey, which again came down to 27.1% during the year 2 survey.

In the linear mixed effects model, after adjusting for other covariates, the mean height of food insecure girls was shorter by 0.87 cm (P<0.001) compared with food secure girls at baseline. However, during the follow up period on average, the heights of food insecure girls increased by 0.38 cm more per year compared with food secure girls (P<0.066). However, the mean height of food insecure boys was not significantly different from food secure boys both at baseline and over the follow up period. Over the follow-up period, adolescents who live in rural and semi-urban areas grew significantly more per year than those who live in the urban areas both for girls (P<0.01) and for boys (P<0.01).

**Conclusions:**

Food insecurity is negatively associated with the linear growth of adolescents, especially on girls. High rate of childhood stunting in Ethiopia compounded with lower height of food insecure adolescents compared with their food secure peers calls for the development of direct nutrition interventions targeting adolescents to promote catch-up growth and break the intergenerational cycle of malnutrition.

## Introduction

Linear growth during adolescence is faster than in any other period of human growth after birth with the exception of the first year of life. As a transitional period between childhood and adulthood, adolescence provides an opportunity to prepare for a healthy productive and reproductive life. Puberty is a dynamic period of growth during adolescence characterized by rapid changes in body composition, shape and size, all of which are distinct for boys and girls. The onset of puberty approximately matches with a skeletal (biological) age of nearly 11 years in girls and 13 years in boys [1,2]. On average, girls pass through each stage of puberty earlier than boys. The timing and duration of this pubertal development is influenced by a number of factors, including genetic characteristics, body composition, physical activity and diet [3-7]. Nutritional status and heavy exercise were identified to be the two major influences on the linear growth of adolescents [8]. However, in food insecure environments, it is hardly possible to fulfill the nutritional requirements of adolescents for healthy growth. Food insecurity is prevalent among adolescents in Jimma, Ethiopia [9-11]. Evidence shows that food insecurity is associated with poor development and morbidity in children [12,13], morbidity [9,14,15] and poor subsequent dietary habits [16] in adolescents. Food-insecure and stressed adolescents are likely to alter their dietary behavior in ways that increase the risk of stunting [16,17]. It has been documented that even stunting that occurs soon after birth can have an impact on adolescent height [18] with a subsequent permanent negative effect on final height. However; growth spurts during adolescence can compensate for earlier stunted growth and provide an opportunity for catch-up growth before final height is attained.

Although childhood stunting is highly prevalent in Ethiopia in general and in the region where this study was conducted in particular [19], there is little research that investigated linear growth during adolescence. A cross-sectional study from Northern Ethiopia documented that 26.5% of adolescent girls were stunted [20]. However; this study did not have data on boys and did not examine the effect of food insecurity on growth. Although adolescents in Jimma zone suffer a number of negative health consequences of food insecurity (9, 15), the effect of food insecurity on linear growth has not been examined. To the best of our knowledge there was no study that examined the growth patterns of adolescents by food security status. This study aimed to determine the effect of food insecurity on the linear growth (height) of adolescents in southwest Ethiopia. We hypothesize that food insecure adolescents are likely to have lower growth (height) over two years follow up period compared to their food secure peers.

## Methods

### Study sample

Data for this study comes from the Jimma Longitudinal Family Survey of Youth (JLFSY) which followed a randomly selected sample of youth starting at ages 13–17 for approximately 5–6 years. The survey began in 2005 and sampled households and adolescents within households from six neighborhoods in Jimma Town (a zonal city of approximately 120,000 inhabitants), three nearby towns, and 18 rural “kebeles” (villages) immediately surrounding the towns. The study rural districts included a coffee growing area (altitude of 1911 meters), a highland vegetable growing area (altitude of 2300 meters), and a lower lying plain area dedicated to grains and other food crops (altitude of 1795 meters).

A street-by-street enumeration of all households was conducted to compile complete list. This generated a list of 5795 households which gave a sampling frame for random selection of 3,700 households. A two-stage sampling plan was used to select the target sample of adolescents. Households were classified into urban (Jimma City), semi-urban (Serbo, Dedo and Yebbu Towns) and six rural “kebeles” (two in the vicinity of each of the three small towns). At the first stage, households were randomly sampled with the sample size in each “kebele” determined by the relative proportion of the study population in the “kebele” and the overall target sample size. In the second stage, one adolescent (a boy or a girl) was randomly selected from each household using a Kish Table [21]. Using this sampling strategy, a total of 1059 boys and 1025 girls were interviewed in round one. The same adolescents were followed and interviewed at one year interval for the subsequent follow up surveys.

### Measurements

Structured household and adolescent level questionnaires were used to collect data. The questionnaires were interviewer-administered and translated into Amharic and Oromifa languages and checked for consistency by other persons who speak both languages and English both at baseline, year 1 and year 2 of the survey. The household questionnaire included a household registry that collected socio-demographic information on all current resident and non-resident household members including information on their income and food security status. The heads of households responded to the household questionnaire. The adolescent questionnaire focused on issues related to adolescents’ experiences of food insecurity, education, health and anthropometric measurements. The interview was conducted in a private place by an interviewer of the same sex as the adolescent respondent after the household interview was completed.

The interviewers received one week of intensive training prior to the pre-test and an additional week of training with the final version of the questionnaire before beginning the actual interviews. Supervisors kept track of the field procedures and checked the completed questionnaires every day to ensure accuracy of the data collected and the research team supervised the field team every week.

Adolescent food insecurity was measured using a four item index adopted from household food security questionnaires used in developing countries [22-24] by modifying the items that could be used at an individual adolescent level. The details of the methods are described elsewhere [9]. To summarize briefly, adolescents were asked whether in the last three months they (1) had ever worried about having enough food, (2) had to reduce food intake because of shortages of food or money to buy food, (3) had to go without having eaten because of shortage of food or money to buy food and (4) had to ask outside the home for food because of shortage of food or money to buy food. A “Yes” response to any of the food security questions was labeled to have score of “1” and “No” was labeled to have score “0”. The values were summed to produce a food insecurity index. The index of food insecurity is defined as the number of items with a positive answer. The index was dichotomized as “food insecure” for adolescents having a value of 1 and above and “food secure” for those who had a value of 0. The index has high internal consistency (Cronbach’s Alpha=0.81), which is above the cut off for reliability [25].

All data on background characteristics, height, and food insecurity were collected at the baseline survey. Height was repeated in the subsequent two rounds of the survey, which were conducted one year apart. Heights of adolescents were measured to the nearest 0.1 cm by trained 12 grade complete data collectors using a stadiometer (SECA, Hannover Germany). The height of each adolescent was measured without shoes. Height for age z-scores were calculated using WHO Anthro-Plus software [26].

We used repeated measures of adolescent food insecurity and height over the 2 follow up surveys and background characteristics from the baseline survey including: place of residence, household income and baseline height for age z-scores as predictor variables. Household income was divided into tertiles coded as “high”, “middle” and “low”.

At each round of the survey the data collection was conducted from August to February. Data collectors were interviewed at similar season of the previous survey as the data collection process was organized in a cyclic and regular manner defining the movement from one study site to the other.

### Statistical analyses

Data were analyzed using SAS version 9.2. The anthropometric data were checked for normality and outliers. First, exploratory analyses were carried out to identify average and subject specific evolution of height over time. The subject specific profile plots suggested that the height of adolescents varied at baseline as well as over time for each subject implying presence of considerable between and within variation. As the repeated measurements of heights taken from each subject over time are correlated, the commonly used methods like linear regression are not appropriate. There is a need for models which can take the correlation into account, combining both the fixed and random effects. To examine differences in heights within individual subjects over the follow up period, a mixed effects(fixed effects and random effects) model with a random intercept and a random slope was fitted with restricted maximum likelihood estimation method using the procedure ‘proc mixed’. The fixed effects describe a population intercept and population slopes for a set of covariates, which include exposures and potential confounders. Random effects describe individual variability in height and changes over time. By considering individual random slopes and intercepts, this model allows to examine the influence of covariates on the change in height over time.

Based on stepwise likelihood ratio test, food insecurity and baseline height for age z-score, time (round of follow-up), age at baseline, household income and place of residence were used as covariates in the mean structure. To determine within subjects effect (growth over time), we included an interaction term of time of follow up with food insecurity, household income and place of residence in the models, while the main effects were used to show the effects at baseline.

Because of the correlated nature of the data, a correlation structure was specified for the measures within individuals. Some commonly used structures, namely, compound symmetric and unstructured, were used to model the covariance structure of repeated measures within subjects. We used unstructured variance matrix for girls, and compound symmetry variance (CS) matrix structure for boys as parameters of the random intercept could not be estimable due to over parameterization in the case of boys.

We stratified the analysis by sex as we expected differences in linear growth between boys and girls. As the growth of boys was not linear, we considered the quadratic effect of the time of follow up in the models. The introduction of the main quadratic time effect into the model improved the model fit based on the maximum likelihood ratio test. However, the interaction of the quadratic time effects with other covariates such as food insecurity, place of residence and income did not have any significant effect on the model fit and hence dropped from the model. All tests were two-sided and P<0.05 was considered statistically significant. We report the parameter estimates and standard errors (SE).

The study was approved by the Ethical Review Boards of both Brown University (USA) and Jimma University (Ethiopia). Informed verbal consent was obtained both from the parents and each respondent before the interview or measurement as approved by the ethical review committees which followed and documented the study process through supervisory visits.

## Results

Overall, a total of 1431 adolescents were followed for three rounds that are one year apart spanning a total follow up period of two years starting 2005. Nearly half (49.2%) were females and the rest (50.8%) were males at baseline. Analysis of socio-demographic characteristics by food security status at baseline is presented in Table 1. It was observed the proportion of food insecure adolescents was significantly high among urban adolescents (23.5%) compared to (20.2%) in the semi-urban areas and 17.9% in the rural areas (P=0.028). Similarly, the proportion of food insecure adolescents was 23% and 21.8%, respectively for low and middle income tertiles compared to 16.8% for high income tertile(P<0.001).

**Table 1:** Association between food security and socio-demographic variables at baseline

**Variables**	**n**	**Food security status**		**P**
		**Food secure(n=1656)**	**Food insecure(n=428)**	
Age(years)				
13	458	76.2	23.8	0.184
14	479	81.8	18.2	
15	479	81.0	19.0	
16	386	77.7	22.3	
17	282	80.4	19.6	
Place of residence				
Urban	746	76.5	23.5	0.028
Semi-Urban	589	79.8	20.2	
Rural	749	82.1	17.9	
Household income tertiles				
low	687	77.0	23.0	0.011
Middle	702	78.2	21.8	
High	695	83.2	16.8	

With regard to exposure of adolescents to food insecurity over time, 15.9% of the girls and 12.2% of the boys (P=0.018) were food insecure both at baseline and year 1 survey, while 5.5% of the girls and 4.4% of the boys (P=0.331) were food insecure in all the three rounds of the survey. In general, a significantly (P=0.045) higher proportion (40%) of girls experienced food insecurity at least in one of the survey rounds compared with boys (36.6%), Table 2.

**Table 2:** Baseline characteristics and repeated measures adolescents who were followed for all the three rounds of survey by sex

**Characteristics**	**Girls**	**Boys**	**N**
Adolescents interviewed (%)			
Baseline	49.2	50.8	2084
Year 1	48.0	52.0	1911
Year2	44.4	55.6	1431
Age of adolescents in years at baseline (%)			
13	47.2	52.8	458
14	48.2	51.8	479
15	52.8	47.2	479
16	49.7	50.3	386
17	47.0	53.0	281
Place of residence (%)			
Urban	52.8	47.2	746
Semi-urban	48.9	51.1	589
Rural	45.8	54.2	749
Baseline household income tertile (%)			
Low	46.9	53.1	687
Middle	47.6	52.4	702
High	53.1	46.9	695
Food insecure (%) ^†^			
Baseline*	25.5(n_1025_)*	15.8(n_1059_)	2084
Both baseline and year 1	15.9(n_917_)*	12.2(n_994_)	1911
All the 3 rounds	5.5(n_635_)	4.4(n_796_)	1431
At least in one of the 3 rounds^ƒ^	40(n635)*	36.6(n796)	1431
Mean (±SD) height (cm)			
Baseline	154.0(7.7)	157.3(11.7)	2084
Year 1	156.9(6.8)	161.5(10.8)	1911
Year 2	158.9(5.7)	169.8(7.0)	1431
Mean (±SD) HAZ^©^ for age z-score at baseline	−0.91(.99)	−1.05(1.2)	2084

^†^The percentages refer to only those who are food insecure and are calculated from sex specific column totals for each follow up year.

^ƒ^Adolescents who were food insecure at least in one of the rounds of the three surveys.

*P<0.05, SD = Standard Deviation.

^©^Height for age Z- Score.

As presented in Table 3, analysis of the trend of food insecurity over the follow period showed doubling of the proportion of adolescents with food insecurity from the baseline (20.5% to 48.4%) on the year 1 survey, which decreased to 27.1% during the year 2 survey.

**Table 3:** Trend of food insecurity and proportion of adolescents with height for age z-scores below −1 by food security and round of follow up

**Variables**	**Food insecurity status**		**P**	**Total**
	**Food secure**	**Food insecure**		
	**n (%)**	**n (%)**		
Round of survey				
Baseline	1656(79.5)	428(20.5)	<0.001	2084
Year 1	987(51.6)	924(48.4)		1911
Year 2	1043(72.9)	388(27.1)		1431
Height for age below -1Z score of the WHO reference				
Baseline				
Yes	747(46.1)	198(49.3)	0.260	915
No	838(53.9)	204(50.7)		1042
Year 1				
Yes	419(44.7)	435(50.8)	0.010	854
No	519(53.3)	422(49.2)		941
Year 2				
Yes	496(47.4)	176(45.0)	0.430	672
No	551(52.6)	215(55.0)		766

**Figure 1:**
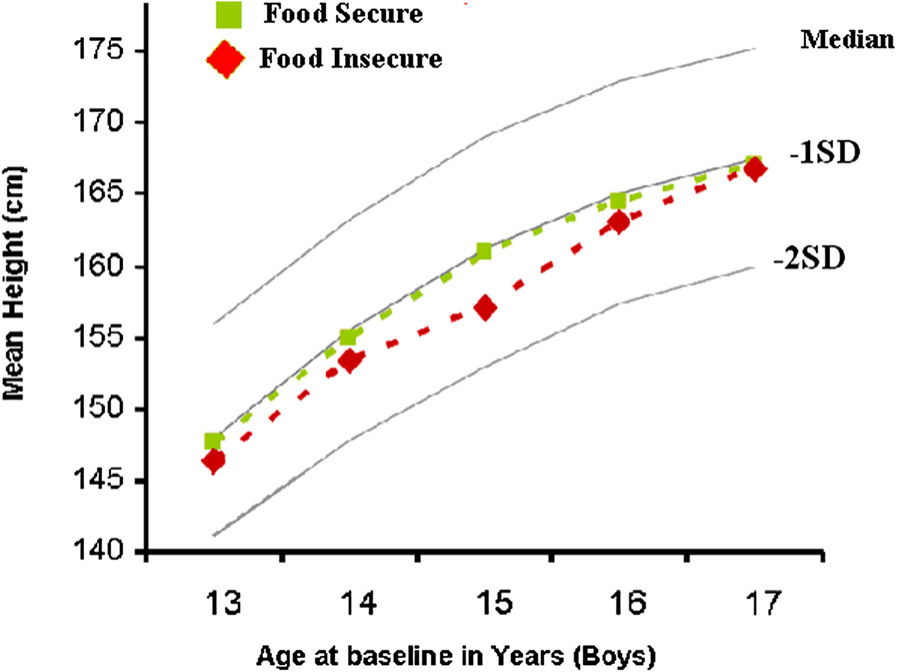
Mean baseline height of adolescents boys by age and baseline food security status compared to the WHO reference.

**Figure 2:**
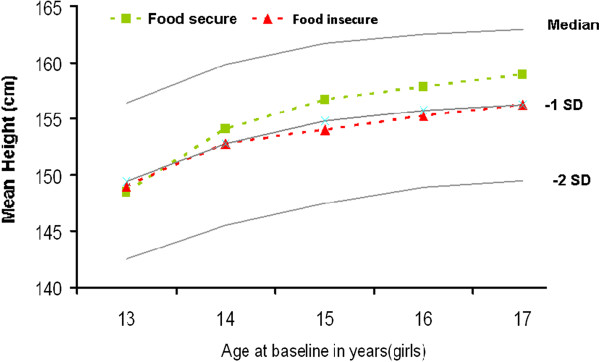
Mean baseline height of adolescent girls by age and baseline food security status compared to the WHO reference.

Figure 1 shows the average baseline heights of adolescent boys by age and baseline food security status compared to the WHO references for their age. Food insecure boys had their mean height on average 2.22 cm below the heights corresponding to –1SD of the WHO reference for their age, while food secure boys had mean height on average 0.32 cm below the heights corresponding to -1SD of the WHO reference. Similarly, as shown in Figure 2, the mean baseline heights of food insecure girls was on average 1.16 cm below the heights corresponding to -1SD of the WHO reference, while that of food secure girls was 1.26 cm above the heights corresponding to -1SD of the WHO reference.

**Figure 3:**
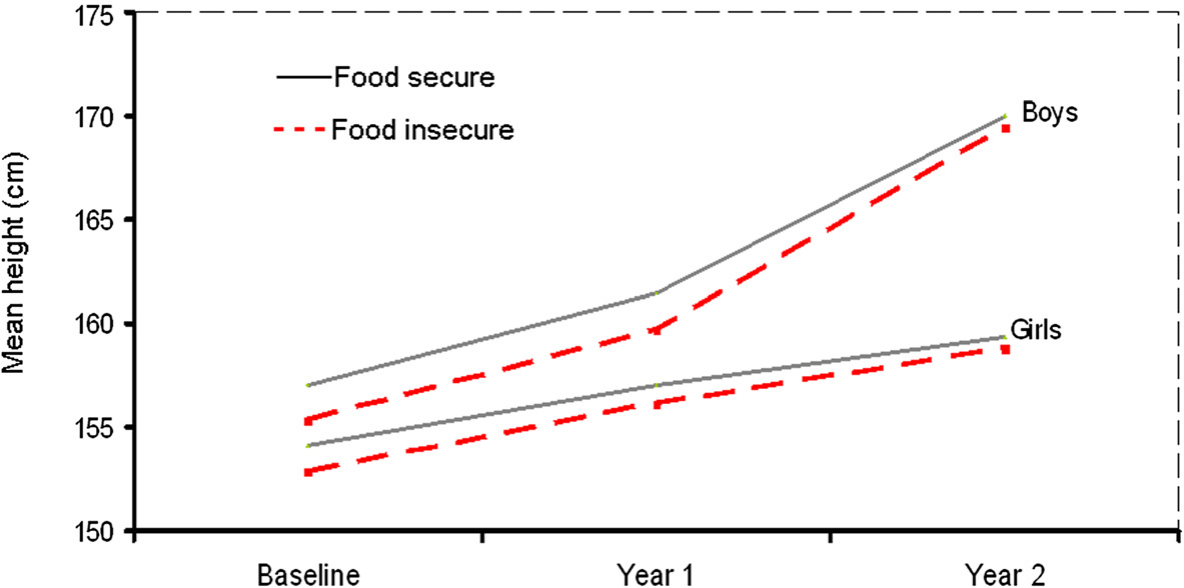
Growths of adolescents over the two years follow up period by food security status and sex.

The average growth of adolescents over the three follow-up surveys is shown in Figure 3. Both food insecure boys and girls had shorter height than their food secure counterparts at all three measurements. In both boys and girls, the difference between food secure and food insecure decreased over the years although it did not disappear fully.

As presented in Table 4, linear mixed effects model showed that food insecurity was negatively associated with height of girls baseline (ß= −0.87, P<0.0001). At baseline, food insecure girls were on average shorter by 0.87 centimeters (cms) compared with food secure girls after controlling for demographic and economic covariates. However, over the follow up period, food insecure girls grew on average by 0.38 cms more per year than food secure girls (ß =0.38, P=0.0663). Other factors positively associated with growth among girls were time (follow up year) and age at baseline. On average, when girls differed with an age of 1 year at baseline, their mean heights differed by 3.57 cms at baseline (ß =3.57, P<0.001). The height of girls increased by 3.44 cms for a unit increase in the follow up year (ß =3.44, P<0.001).

**Table 4:** Parameter estimates and standard errors from linear mixed effects model predicting linear growth (height increase) of girls over the follow up period

**Effect (n=1431)**	**Estimate**	**SE**	***P***
***Fixed Effects***			
Intercept	110.260	0.9641	<.0001
Food Insecurity	−0.8689	0.1907	<.0001
Food Insecurity x Time	0.3750	0.2040	0.0663
Height for age z-scores at baseline	7.3941	0.0758	<.0001
Time	3.4408	0.2325	<.0001
Age of girls at baseline	3.5715	0.0627	<.0001
Household Income Tertile			
High (Reference)			
Middle	0.2259	0.2193	0.3032
Middle x time	0.4781	0.2993	0.1105
Low	0.6734	0.2275	0.0031
low x time	0.2218	0.3090	0.4729
Place of Residence			
Urban (Reference)			
Semi-urban	0.2694	0.2109	0.2016
Semi-urban x time	0.9145	0.2877	0.0015
Rural	0.2242	0.2228	0.3146
Rural x Time	1.1813	0.3007	<.0001
***Random effects***			
Variance of Random Intercept	2.9134	0.5852	
Variance of Random Slope	13.9245	0.7515	
Covariance of Random Intercept and Slope	5.6652	0.5789	
Variance of measurement errors (residuals)	12.0751	0.4551	

Restricted maximum likelihood (REML) method was used in estimating the parameters.

SE = Standard error.

Time = follow up rounds.

While place of residence did not have any effect on the baseline heights of girls, there was a significant difference in their growth by place of residence over the follow up period.

Compared to urban girls, the growth rate of rural girls was greater by 1.18 cms for a unit increase in the follow up year (P<0.0001), while, those in the semi-urban areas grew by 0.92 cms more for a unit increase in the follow up year compared to urban girls (P=0.0015), after adjusting for all covariates. Baseline height for age z-score was also positively associated with growth in girls (P<0.0001). For a unit increase in baseline height for age z-score, the height of girls increased by 7.39 cms on average.

Results of linear mixed effects model for boys (Table 5) showed that on average when boys differed with an age of 1 year at baseline, their mean heights differed by 3.95 cms (ß=3.95, P<0.001). Food insecurity did not have a significant effect on the height of boys both baseline (ß=−0.34, P=0.3210) and over the follow up period (ß =0.14, P=0.6249). At baseline, food insecure boys were on average shorter by 0.34 centimeters (cms) after controlling for all other covariates. However, over the follow up period, food insecure boys grew by 0.14 cms more per year than food secure boys.

**Table 5:** Parameter estimates and standard errors from linear mixed effects model predicting linear growth (height increase) of boys over the follow up period

**Effect (n=1431)**	**Estimate**	**SE**	***P***
***Fixed Effects***			
Food Insecurity	−0.3382	0.3405	0.3210
Height for age z-scores at baseline	6.6557	0.0911	<.0001
Food Insecurity X Time	0.1420	0.2904	0.6249
Time	1.3737	0.4000	0.0006
Age of boys at baseline	3.9514	0.0804	<.0001
Household Income Tertile			
High (Reference)			
Middle	−0.3571	0.3695	0.3343
Middle X time	0.6131	0.3522	0.0822
Low	0.1416	0.3864	0.7140
low X time	−0.1378	0.3663	0.7069
Place of Residence			
Urban (Reference)			
Semi-urban	−0.3955	0.3546	0.2651
Semi-urban X time	0.9695	0.3396	0.0040
Rural	−0.6366	0.3717	0.0873
Rural X Time	1.1587	0.3546	0.0011
Quadratic time effect (Time^2^)	1.9663	0.1565	<.0001
***Random effects***			
Variance	14.6274	1.5378	
CS	−5.1433	0.8679	
Variance of measurement errors (residuals)	11.4764	0.4959	

Restricted maximum likelihood (REML) method was used in estimating the parameters.

Compound symmetry (CS) variance matrix structure was used to avoid over parameterization of the estimates.

SE= Standard error.

Time = follow up rounds.

Other factors that were positively associated with growth among boys were time (follow up year) and baseline height for age z-score. Over the follow up years, the height of boys increased by 1.37 cms for a unit increase in the follow up year (ß =1.37, P<0.0001). Similarly, for a unit increase in the height for age z-score at baseline, height of boys increased by 6.66 cms (ß =6.66, p<0.001). As the mean growth plot of boys over the follow up period did not follow a linear pattern, we used a quadratic time effect in the model which was positively associated with growth (ß =1.97, P<0.0001). Introduction of the quadratic time effect into the model improved the model fit significantly (P<0.001) based on the likelihood ratio test.

Similar to the pattern observed in girls, boys had also different trajectory of growth by residence. Over the follow up period, for a unit increase in the follow up year, rural boys grew by 1.16 cms per year than their peers in the urban areas (P<0.001), while semi-urban boys grew 0.97 cms taller per year than their urban peers (P=0.004).

## Discussion

This study longitudinally examined the effect of food insecurity on linear growth of adolescents in the southwest Ethiopia, where food insecurity is a pervasive phenomenon [9-11]. We found that food insecure girls were shorter by nearly 1 cm (0.87 cm) compared with secure girls at baseline. Reports from the same cohort also showed that girls suffered from chronic food insecurity [9,11], morbidity [15] school absenteeism [27] and low dietary diversity more than their boy counterparts. The fact that food insecurity was associated with a significant negative effect on the baseline height of girls might be related to male biased intra-household buffering of adolescents from food insecurity. A similar male gender bias in the intra-household distribution of food and other resources have been reported from Ethiopia [9] and other developing countries [28-31] indicating that girls are less favored in the resource constrained environments.

Food insecurity through its effect on adequate nutrient intake could hinder occurrence of catch up growth to rectify the height deficits accrued from childhood. In a food insecure situation, adolescents use food based coping strategies to survive with food shocks. These coping strategies could range from the reversible insurance strategies such as consuming low quality cheap foods, reducing the size or frequency of consumption, searching for food in socially unacceptable ways and food anxiety [23,32]. Use of any of the coping strategies could play against the nutrient intakes of adolescents potentially leading to growth retardation. Poor dietary practices in food constrained situations are related to dietary behaviors adapted in response to food shortages [32,33]. Stunted growth had been reported to be associated with poor dietary practices [2,4,5].

Recent research findings, in Guatemala indicated that stunting in early childhood can have long-term effects on cognitive development, school achievement, and economic productivity in adulthood and maternal reproductive outcomes [34]. Adolescence is the last chance for catch up of linear growth to achieve genetic potential in height [35,36]. As a result of this rapid growth, adolescents need better nutritional care. Adverse circumstance such as food insecurity during this period would interfere the occurrence of catch up growth leading to a short adult stature. This shortness is associated with poor physical and intellectual productivity in both boys and girls. It was documented that 1% decrease in height leads to 1.4% decrease in productivity [37-39]. Empirical evidences from Ethiopia also showed that nutritional status affects agricultural productivity in adults [40]. In this study, food insecurity had significant negative effect on the growth especially in girls. Short girls are likely to have difficulties with labor and child birth [41]. In the long run, they are also likely to have low birth weight babies which will growth below the standard closing the loop of intergenerational of cycle of malnutrition [42].

Stunting during childhood can be reversed with appropriate interventions [43]. There is a potential that children who suffered childhood stunting could show catch up growth [35,44] although it is not a complete catch up as a complete catch growth may require trans-generational catch-up [44]. A study from Senegal reported that children stunted during childhood caught up with their genetic potential, although the height difference between stunted and non-stunted children continued to persist [35]. It has been shown that the four phases of human growth (fetal, infancy childhood and pubertal) are interlinked. Inadequate height gain during the childhood phase may trigger more height gain during the puberty phase and catch-up growth during puberty is likely to occur if the child has experienced malnutrition in childhood [44,45]. Our results showed that regardless of the faster growth of food insecure girls and boys during the two years follow up period, they did not catch up their food secure peers indicating that the catch up growth is not adequate. Unlike the catch up growth in younger children (Type A), where the growth increases up to 4 times the mean growth velocity for chronological age, the type of catch up growth that occurs during adolescence is type B, where a delay in growth and somatic development persists when growth restriction ceases and growth continues for longer than usual to compensate for the growth deficit [46]. However, in this study, we used adolescents who were 13–17 years who had already elapsed their growth spurt ages, especially in the case of girls [1,2], which is one of our limitations. Despite the fact that growth might continue for longer period to compensate for growth deficits of food insecure adolescents, the fact that they are already beyond the age of growth spurt, the slow increase in height in Type B catch up growth and the pervasive nature of food insecurity in the study community may jeopardize the possibility of full catch up in both boys and girls.

Adult short stature results from nutritional deficit at the different phases of growth rather than just in a single phase [45]. Evidence shows that children with faster linear growth in infancy and childhood had less height gain between ages during adolescence [45,47]. Childhood stunting is highly prevalent in Ethiopia [19,48] and the last chance for curbing the consequences of malnutrition on linear growth is adolescence period. Our results imply that in food insecure situations, adolescents need to be targeted in nutrition programs in addition to intervention that are in place during early childhood to promote catch up growth and prevent them form growing into an adult with a short stature. Chronic malnutrition during early childhood is responsible for widespread stunting and adverse health and social consequences throughout the life span. Although this is best prevented during childhood [44] to improve access to food could benefit adolescents as well. Key interventions to promote adolescent growth such as prevention of anemia through iron supplementation and or deworming, school feeding programs and prevention of teenage pregnancy and improving the nutritional status of girls before they enter pregnancy could help to break the intergenerational cycle of malnutrition [49]. In addition to the above, food security intervention targeting adolescents’ should be considered.

For both boys and girls, adolescents in the rural and in the semi-urban areas grew more per years than those in the urban areas. This might be related to the lower frequency of food insecurity prevailing in these areas compared to the urban areas as presented in Table 1.

This study used longitudinal data to demonstrate the effect of food insecurity on growth of adolescents. We acknowledge limitations in interpreting the results of this study. During the follow up period, large number of adolescents was lost to follow up (31.3% of the baseline sample), although this has been taken into consideration during the sample size calculation. We considered in our analyses if this attrition has affected the results in an important way. Most of the adolescents lost were females and older age groups and there was no difference in baseline food insecurity between adolescents that are still in the study and those who were lost to follow up (19.5% VS 22.5%, P=0.081). Entering the effect of adolescents lost to follow up into the multivariable model showed that there was no significant effect in the results. Use of self reported food insecurity questions could also lead to social desirability biases. However as the food security scales are validated in developing countries, it is expected that this problem is minimal. Errors during anthropometric measurements are also possible and could lead to measurement bias. However, in this study, the data collectors were intensively trained on anthropometric measurement procedures and they were also supervised during data collection.

## Conclusions

Food insecurity is negatively associated with the linear growth of adolescents, especially on girls. Although on average food insecure boys and girls grew faster over the follow up period, they did not catch with their food secure peers at the end of the two years follow up indicating that the catch up is not adequate. The results indicate that as catch up growth takes longer time, food insecure adolescents towards the end of their growth years may not be able to catch up. High prevalence of childhood stunting in Ethiopia compounded with the negative effect of food insecurity on adolescents growth calls for the development of direct nutrition interventions targeting adolescents to promote faster catch-up growth and break the intergenerational cycle of malnutrition. Inadequate growth (stunting) is the reflection of socioeconomic development demanding combination of different types of policies and programmes for its solution. Strategies for reducing and preventing stunting need to involve a combination of macro-economic policies and more targeted interventions. Promoting gender equality and the status of women and girls through effective behavior change communications at grassroots level needs to be considered. Further research will help to understand these linkages as a way to identify the most effective strategies for reducing adolescent malnutrition in the socio-cultural environment of the study community.

## Competing interests

The authors declare that they have no competing interests.

## Authors’ contributions

The authors’ responsibilities were as follows: DL, CH, TB & AG: Designed and supervised the study and ensured quality of the data and made a substantial contribution to the local implementation of the study and PK, CH, WK & DL assisted in the analysis and interpretation of the data. All authors critically reviewed the manuscript. TB, the corresponding author did the analysis & drafted the manuscript and had the responsibility to submit the manuscript for publication. All authors read and approved the final manuscript.
